# Cloning and characterization of a novel alternatively spliced transcript of the human CHD7 putative helicase

**DOI:** 10.1186/1756-0500-3-252

**Published:** 2010-10-06

**Authors:** Christian Colin, Flávia S Tobaruella, Ricardo G Correa, Mari C Sogayar, Marcos A Demasi

**Affiliations:** 1 Chemistry Institute, University of São Paulo, Biochemistry Department, São Paulo, 05508-000 SP, Brazil; 2 Dana-Farber Cancer Institute, Department of Cancer Biology, Harvard Medical School, One Jimmy Fund Way, Boston, MA 02115, USA; 3 Burnham Institute for Medical Research, La Jolla, CA 92037, USA

## Abstract

**Background:**

The *CHD7 *(Chromodomain Helicase DNA binding protein 7) gene encodes a member of the chromodomain family of ATP-dependent chromatin remodeling enzymes. Mutations in the *CHD7 *gene are found in individuals with CHARGE, a syndrome characterized by multiple birth malformations in several tissues. CHD7 was identified as a binding partner of PBAF complex (Polybromo and BRG Associated Factor containing complex) playing a central role in the transcriptional reprogramming process associated to the formation of multipotent migratory neural crest, a transient cell population associated with the genesis of various tissues. *CHD7 *is a large gene containing 38 annotated exons and spanning 200 kb of genomic sequence. Although genes containing such number of exons are expected to have several alternative transcripts, there are very few evidences of alternative transcripts associated to *CHD7 *to date indicating that alternative splicing associated to this gene is poorly characterized.

**Findings:**

Here, we report the cloning and characterization by experimental and computational studies of a novel alternative transcript of the human *CHD7 *(named CHD7 CRA_e), which lacks most of its coding exons. We confirmed by overexpression of CHD7 CRA_e alternative transcript that it is translated into a protein isoform lacking most of the domains displayed by the canonical isoform. Expression of the CHD7 CRA_e transcript was detected in normal liver, in addition to the DU145 human prostate carcinoma cell line from which it was originally isolated.

**Conclusions:**

Our findings indicate that the splicing event associated to the CHD7 CRA_e alternative transcript is functional. The characterization of the CHD7 CRA_e novel isoform presented here not only sets the basis for more detailed functional studies of this isoform, but, also, contributes to the alternative splicing annotation of the *CHD7 *gene and the design of future functional studies aimed at the elucidation of the molecular functions of its gene products.

## Background

The *CHD7 *(Chromodomain Helicase DNA binding protein 7) gene encodes a member of the chromodomain family of ATP-dependent chromatin remodeling enzymes. In 2004, *CHD7 *was described as the major gene involved in the CHARGE syndrome [1], a complex genetic disorder related to multiple birth malformations and functional disorders, including ocular coloboma (C), heart disease (H), choanal atresia (A), retarded growth and/or anomalies of the central nervous system (R), genito-urinary defects and/or hypogonadism (G), and ear anomalies and/or deafness (E) [2]. *De novo *mutations in the *CHD7 *gene, especially nonsense and frameshift mutations, are found in approximately 60% of the individuals with CHARGE [1-4]. Embryonic lethality at E10.5-E11.5 in mice which are homozygous for null mutations in *Chd7 *support the haplo-insufficiency model as the most likely mechanism involved in this syndrome. Additionally, mice which are heterozygous for null mutations in *Chd7 *recapitulate many of the traits found in individuals with CHARGE, including defects in the eye, heart, choanae, genitals and inner ear [5].

Some lines of evidence suggest that CHD7 is involved in transcription control through ATP-dependent chromatin remodeling [6,7]. Firstly, members of the chromodomain family share a unique combination of functional domains. In CHD7, these domains are the two N-terminal chromodomains thought to mediate binding to methylated histones [6], two SWI2/SNF2-like ATPase/helicase domains, a DNA and/or modified histones binding domain [6], and two BRK (BRM and KIS) domains of unknown function [8]. Secondly, it was recently demonstrated that CHD7 associates with PBAF, a chromatin-remodeling subcomplex of the SWI/SNF (Swich 2/Sucrose Non-fermentable 2) family, and it is essential for the activation of the transcriptional program associated with the formation of multipotent migratory neural crest, a transient cell population with a multilineage differential potential [7]. This cell population is associated with the genesis of various body structures including the peripheral nervous system, pigment cells, craniofacial skeleton and cardiac structures [7,9,10]. Therefore, it is assumed that the mechanistic link between the CHARGE syndrome pathogenesis and the CHD7 protein would be its potential role in regulating embryonic development by affecting chromatin structure and gene expression.

Some important questions remain open regarding *CHD7 *function. One of these questions is related to alternative splicing associated to the *CHD7 locus. CHD7 *is a relatively large gene containing 38 annotated exons and spanning approximately 200 kilobases of genomic sequence. Although it is expected that large genes containing several exons, like *CHD7*, would produce various alternative transcripts [11], to date, very few examples are available of alternative transcripts associated to this gene, suggesting that alternative splicing associated to the *CHD7 *gene is underestimated and far from being annotated. Although alternative splicing events related to CHD genes remains largely unexplored, Duplin, a CHD8 isoform generated by alternative splicing with a crucial role in embryogenesis, is an example of the complexity of gene products that can be potentially produced from *CHD loci *[12]. In this context, characterization of alternatively spliced transcripts related to the *CHD7 locus *is fundamental for the design of future functional studies.

In the present study, we have characterized the exon-intron structure of a novel *CHD7 *alternative transcript and its expression profile at mRNA level in some human cell lines and normal human tissues. This alternative transcript misses most of *CHD7 *coding exons, being translated as a CHD7 isoform lacking most of the domains presented in the canonical isoform. We named this novel transcript and its encoded protein isoform CHD7 CRA_e, following the existing *CHD7 *isoform nomenclature and will keep to this nomenclature in this study.

## Methods

### Cell lines

The following human cell lines: DU145 prostate carcinoma, HepG2 hepatocarcinoma, transformed 293 T kidney embryonic, Skmel-25 malignant melanoma, NCI-H1155 lung carcinoma, IM-9 B transformed lymphoblast, SAOS 2 osteosarcoma and HeLa cervix adenocarcinoma were cultured as previously described [13] and recommended by [14].

### Reverse transcription and polymerase chain reaction

In order to obtain the coding sequence of the canonical isoform of the *CHD7 *gene, total RNA samples from diverse tumor cell lines were purified according to the Chirgwin procedure [15]. Poly A^+ ^RNA was isolated from total RNA with the PolyAttract mRNA isolation kit (Promega, Madison, WI) and employed for cDNA synthesis.

cDNA was synthesized using 1 μg of poly A^+ ^RNA, oligodT_12-18 _and the Superscript III reverse transcriptase (Invitrogen, Carlsbad, CA), according to manufacturer's instruction. PCR reactions were carried out in 25 μl and the reactions mixtures contained 1 μl of a 1/20 dilution of the cDNA preparation, 1× Tuning Buffer^® ^(Eppendorf, Westbury, NY), dNTPs (0.5 mM each), 0.5 μM of each primer and 0.5 U TripleMaster Taq polymerase (Eppendorf, Westbury, NY). The following primers annealing to sequences corresponding to exons 1 and 38 of *CHD7 *were used:

CHD7F forward: 5'-AAAAAGCAGGCTTGGTCCTCGCCACGCGCTCGTGCTCGGGA-3' and CHD7R reverse: 5'-AGAAAGCTGGGTGGGACATCTCTGCATATCATGGGTCACT-3'.

A "Long distance PCR" cycling protocol was employed, as follows: 3 min at 93°C (initial denaturation); 14 cycles of 20 sec at 93°C and 10 min at 68°C; 21 cycles of 20 sec at 93°C and 11 min at 68°C with additional 20 sec of auto-extension at each cycle; 7 min at 68°C (final extension).

To analyze the expression of the *CHD7 *novel splicing variant by RT-PCR, its sequence was used to design a set of primers flanking the splicing site at exons 3 (CHD7 E3 forward 5'-AGTGCTGGGATACCAATGGA-3') and 36 (CHD7 E36 reverse 5'-GGAACCCCCATACAGTCAAA-3'), which would yield a PCR band of approximately 2 kbp. Expression of the long transcript from the *NOTCH2 *gene was evaluated as an internal control using the following primers corresponding to the 5' region of the transcript:

NOTCH2 forward 5'-ACTGTGGCCAACCAGTTCTC-3' and NOTCH2 reverse 5'-CTCTCACAGGTGCTCCCTTC-3', which would yield a PCR band of approximately 300 bp. For this analysis, cDNA was synthesized using Superscript III reverse transcriptase (Invitrogen, Carlsbad, CA), according to manufacturer's instruction and 1.5 μg of total RNA from the DU145 prostate carcinoma cell line and from various human tissues (spinal cord, prostate, kidney, lung, placenta, skeletal muscle and liver) obtained from the Human Total RNA Master Panel II (Clontech, Mountain View, CA). Polymerase chain reactions (final volumes 50 μl) containing 1× Phusion HF Buffer (Finnzymes, Finland), 0.5 mM each dNTP, 0.4 uM each primer, 1 ul of the undiluted cDNAs preparations, and 2 U Phusion Hot Start DNA Polymerase (Finnzymes, Finland) were carried out to detect the novel *CHD7 *alternative isoform and *NOTCH2 *transcripts in the cDNA samples mentioned above. The cycling protocol employed for the detection of CHD7 CRA_e transcript was as follows: 30 sec at 98°C (initial denaturation); 3 cycles of 10 sec at 98°C, 30 sec at 68°C and 12 min at 72°C; 3 cycles of 10 sec at 98°C, 30 sec at 65°C and 12 min at 72°C; 3 cycles of 10 sec at 98°C, 30 sec at 62°C and 12 min at 72°C; 35 cycles of 10 sec at 98°C, 30 sec at 60°C and 12 min at 72°C; and a 15 min at 72°C (final extension). The cycling protocol employed for the detection of *NOTCH2 *transcript was as follows: 4 min at 94°C (initial denaturation); 35 cycles of 30 sec at 94°C, 45 sec at 54°C and 45 sec at 72°C; and a 10 min at 72°C (final extension).

A negative control, without cDNA, was run with each reaction. PCR products were fractionated by agarose gel electrophoresis and visualized under UV light and the digital images were acquired using the D-Transilluminator and MiniBIS gel documentation system (DNR Bio-Imaging Systems, Israel).

### Sequence analysis of the novel CHD7 transcript

In order to characterize the novel *CHD7 *transcript, its coding sequence was re-amplified from a cDNA preparation synthesized using the Superscript III reverse transcriptase (Invitrogen, Carlsbad, CA), according to manufacturer's instructions, and 3 μg of total RNA from DU145 prostate carcinoma cell line. PCR reactions were carried out in 50 μl and the reactions mixtures contained 1 μl of a 1/20 dilution of the cDNA preparation, 1× High Fidelity Buffer^® ^(Eppendorf, Westbury, NY), dNTPs (0.4 mM each), 0.4 μM of each primer and 4 U TripleMaster Taq polymerase (Eppendorf, Westbury, NY). The following primers annealing to sequences corresponding to exons 2 and 38 of *CHD7 *were used: CHD7F forward: 5'-ACCTCAGTGAAGTGAAGCACAGG-3' and CHD7R reverse: 5'-CACACTAGCGTGGAGATTGTCAG-3'. The cycling protocol employed was as follows: 3 min at 94°C (initial denaturation); 1 cycle of 30 sec at 94°C and 12 min at 72°C; 3 cycles of 30 sec at 94°C, 40 sec at 68°C and 12 min at 72°C; 3 cycles of 30 sec at 94°C, 40 sec at 65°C and 12 min at 72°C; 35 cycles of 30 sec at 94°C, 40 sec at 62°C and 12 min at 72°C; and a 15 min at 72°C (final extension).

The approximately 3.3 kpb DNA band was gel purified and subcloned into the pGEM-T Easy vector (Promega, Madison, WI) using the TA cloning system, according to manufacturer's instruction. Three bacterial (*Escherichia coli *XL1 Blue) clones (pGEM-M1, pGEM-M2 and pGEM-M3) were picked and individually grown in liquid LB medium containing 100 μg/ml of ampicilin overnight at 37°C under agitation (250 rpm). Plasmid DNA was extracted from bacterial cultures using the GFX TM Micro Plasmid Prep (GE HealthCare, Piscataway, NJ), according to the manufacturer's instructions. The three clones containing the novel *CHD7 *transcript cDNA were subjected to sequencing using the ABI 3700 sequencer and the BigDye 3.1 sequencing kit (Applied Biosystems, Foster City, CA) at the GaTE (Genomic and Transposable Elements) lab, Biological Institute, University of Sao Paulo. Sequencing was carried out using sequencing primers annealing to *CHD7 *exons 2, 37 and 38. The novel *CHD7 *transcript cDNA sequences (clones M1, M2 and M3) were individually clustered into contigs using the SeqMan II software (DNASTAR, Inc., Madison, WI). The original sequencing files were evaluated for quality using the Trace Quality Evaluation algorithm within SeqMan II. Poor-quality sequences were trimmed and the trimmed cDNA sequences of each clone were assembled into contigs using the SeqMan II assembly process. The contig cDNA sequences of each clone were individually compared to the canonical *CHD7 *transcript reference sequence (NM_017780.2) using the BLASTN program [16] at NCBI [17]. Determination of sequence overlap between the contig cDNA sequences described above and the *CHD7 *canonical transcript reference sequence and *CHD7 *mRNAs and spliced ESTs sequences from the Genbank was performed using the UCSC Genome Browser [18,19]. The contig cDNA sequences were aligned to the February 2009 version of the human genome sequence assembly using the BLAT alignment tool provided by UCSC. The contigs cDNA sequences open reading frames (ORFs) were determined using the ORFinder tool at [20]. Translations of the detected ORFs were submitted to alignment to CHD7 reference protein sequence (Genbank: NP_060250.2) using the BLASTP alignment tool [21].

### Cloning of the CHD7 CRA_e isoform coding sequence into a bicistronic lentiviral expression vector

The CHD7 CRA_e isoform coding sequence from clones M1, M2 and M3 were amplified by PCR from pGEMT-M1, pGEM-M2 and pGEM-M3 vectors and sub-cloned into the p156RRLsinPPTCMVIRESPRE third generation transfer bicistronic lentiviral vector [22] (heretofore referred to as pLV-EGFP). This pLV-EGFP bicistronic vector was kindly provided by Prof. Inder Verma (The Salk Institute, San Diego, California) and further modified in our lab by Dr. Juan Carlos Bustos Valenzuela to add the 5'-XbaI-EcoRV-MluI-NheI-PstI-XhoI-BamHI-3' multiple cloning site. Polymerase chain reactions (final volumes 50 μl) containing 1× HiFi Buffer (Eppendorf, Westbury, NY), 0.2 mM each dNTP, 0.25 uM each primer, 50 ng of the template plasmids, and 2 U Triple Master DNA Polymerase (Eppendorf, Westbury, NY) were carried out to obtain the *CHD7 *novel isoform coding sequences from clones M1, M2 and M3. The following primers were used: VL XhoI IM CHD7 forward: 5'-CCCCTCGAGATGGCAGATCCAGGAATG-3' and VL BamHI IM CHD7 reverse: 5'-CCCGGATCCCTTGAACTGGAACTGGTACTGG-3'. The cycling parameters were as follows: 2 min at 94°C (initial denaturation); 24 cycles of 30 sec at 94°C, 30 sec at 58°C and 4 min at 68°C; and 10 min at 68°C (final extension). The purified PCR products were digested by XhoI and BamHI and sub-cloned into the pLV-EGFP vector digested with the same enzymes.

### Lentivirus particles production and transduction of DU145 cells

Lentivirus particles containing the M1, M2 and M3 cDNA clones of the CHD7 CRA_e isoform and control EGFP lentiviral expression vectors were produced by transient transfection into 293 T cells. Briefly, 2 × 10^6 ^cells were plated in 6-cm diameter Petri dishes 16 h prior to transfection in Dulbecco's modified Eagle medium (DMEM) supplemented with 10% fetal bovine serum (Hyclone, Logan, UT), ampicilin (25 μg/ml), streptomycin (100 μg/ml) and 1.2 g/l of sodium bicarbonate in a humidified atmosphere of 2% CO_2 _in air at 37°C. A sample (total of 5 μg) of plasmid DNA was employed for transfection, as follows: 2.2 μg of the transfer vector plasmid, 1.45 μg of packaging plasmid pMDL (Invitrogen, Carlsbad, CA), 570 ng of the pREV expression vector (Invitrogen, Carlsbad, CA) and 790 ng of the pVSVG envelope plasmid (Invitrogen, Carlsbad, CA). These plasmids were co-transfected into 293 T cells by lipofection using the Lipofectamine 2000 reagent (Invitrogen, Carlsbad, CA) according to the manufacturer's instructions. After 5 h of transfection, the medium was replaced and the conditioned medium containing the pseudo-lentiviral particles were harvested 24, 48 and 72 h after transfection, cleared by low-speed centrifugation and stored at -80°C. For titration of the lentiviral preparations, serial dilutions of the conditioned medium were used to transduce 10^5 ^293 T cells as described elsewhere [23]. The transduction efficiency was estimated by counting EGFP-positive cells under a fluorescence microscope (TE300 Nikon, Japan). The viral titer of each preparation was calculated according to the following formula:

Titer(cfu/ml)=(P×N/100×V)×1/DF,

where P = % EGFP+ cells, N = number of cells at the time of transduction (10^5^), V = volume of dilution used for transduction and DF = dilution factor.

For transduction of DU145 cells, 2 × 10^4 ^cells in suspension were mixed with samples of lentivirus supernatants at a MOI (Multiplicity of Infection) of 10 in the presence of polybrene (10 μg/ml). After mixing, the DU145 cells were plated in 48-well dishes and cultured in the presence of lentivirus for 16 hours. Cells were analyzed for EGFP expression 72 hours after transduction.

### Protein extraction

DU145 cells and the transduced DU145 cells generated as described in the item above (DU145 M1, DU145 M2, DU145 M3 and DU145 EGFP) were plated (1.5 × 10^6 ^cells) in 6-cm diameter Petri dishes and grown overnight. The culture medium was then removed and the cell monolayers were rinsed twice and then scrapped with cold PBSA. The cell suspensions were transferred to microcentrifuge tubes and pelleted for 5 min at 200 × *g *at 4°C. The cells were resuspended in RIPA^+ ^buffer (10 mM Tris-HCl pH 7.5, 1% sodium deoxycholate, 1% NP-40, 150 mM NaCl, 0.1% SDS, 1 mM DTT and 1× protease inhibitors cocktail (GE HealthCare, Piscataway, NJ) and incubated on ice for 15 min. Cell lysates were then homogenized by passing through an insulin syringe several times. Finally, cellular debris was removed by centrifugation (20,000 × *g *for 30 min) at 4°C and the extracts were stored at -70°C. The protein concentration in these extracts was determined using the Bradford assay.

### Western blot analysis

Protein samples (30-40 μg) obtained from cell lysates were fractionated in 6% SDS-polyacrylamide gel electrophoresis. The resolved proteins were electro-blotted onto nitrocellulose membranes (Bio-Rad, Hercules, CA), which were blocked with 5% non-fat milk in TBS buffer containing 0.05% Tween 20, overnight at 4°C. After six washes with TBS/0.05% Tween 20, the membranes were incubated with antibodies against CHD7 (1:400 dilution) (ab31824 - Abcam, Cambridge, UK) in the same buffer containing 5% non-fat milk for 1 h at room temperature. The membranes were washed again and then probed with horseradish peroxidase-conjugated secondary antibodies (Vector Laboratories, Burlingame, CA), in the same buffer, for 40 min at room temperature. PACE (Paired basic amino acid cleaving enzyme) detection using the polyclonal antibody against PACE (sc-20801 - Santa Cruz Biotechnology, Santa Cruz, CA) was used (1:500 dilution) as an internal control. The signals were detected using the ECL-Plus detection system (GE HealthCare, Piscataway, NJ) according to the manufacturer's instructions.

## Results

### Identification of a novel CHD7 alternative transcript by RT-PCR from the human prostate carcinoma cell line DU145

**Figure 1:**
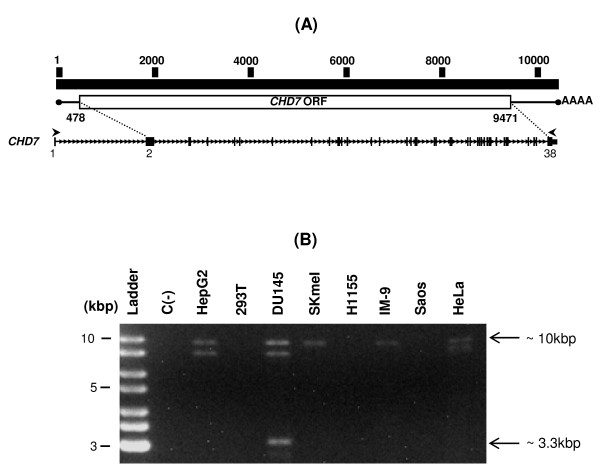
**Identification of a putative novel splice variant of *CHD7***. (A) Representation of the full-length *CHD7 *transcript structure and primers used to amplify it. At the top the nucleotide sequence numbering scheme of the full-length transcript is represented. At the middle is a schematic representation of the full-length *CHD7 *transcript with its ORF indicated as a box (the ORF initial and final nucleotides numbers are indicated below the box). At the bottom the exon-intron structure of *CHD7 *is represented. Exons are represented as black rectangles and intronic sequences as a thin line. The sites of the ORF initial and final nucleotides (dotted lines) and the primers (arrows) annealing to sequences corresponding to exons 1 and 38 of the *CHD7 *transcript sequence (deposited under the accession number NM_017780.2) used for the amplification of the full-length *CHD7 *transcript are indicated. (B) Amplification of the full-length *CHD7 *transcript by RT-PCR. Poly A^+ ^RNA was extracted from the indicated cell lines, and reverse transcription followed by PCR was performed using the *CHD7 *primers set depicted in (A). Aside from the predicted 10 kbp band, additional RT-PCR products of approximately 9 kbp (not indicated) and 3.3 kbp (indicated) were detected. C(-) is the negative control (PCR reaction without cDNA).

In order to clone the full-length *CHD7 *transcript, we designed primers on exons 1 and 38 of *CHD7*, which would yield an RT-PCR product of approximately 10.3 kbp (Figure 1a). RT-PCR reactions using these primers and poly A^+ ^RNA samples from eight human cell lines yielded a DNA band of approximately 10 kbp in most of the samples (Figure 1b). Additionally, a band of approximately 9 kbp was also detected in RNA samples from three cell lines (HepG2, DU145 and HeLa) and another one of approximately 3.3 kbp in the human prostate carcinoma cell line DU145 (Figure 1b).

**Figure 2:**
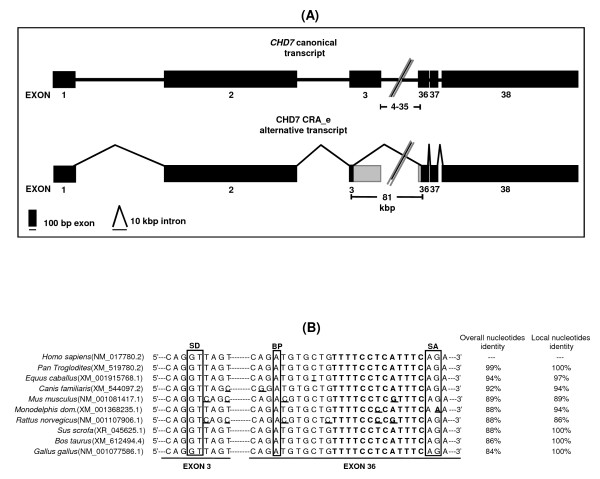
**Analysis of the exon-intron structure of the CHD7 CRA_e novel transcript**. (A) In the upper part of the figure the exon-intron structure of the canonical *CHD7 *gene (accession number NM_017780.2) is represented. Exons 4 to 35 have been omitted for clarity. In the lower part the exon-intron structure of the CHD7 CRA_e alternative transcript deduced from sequencing and annealing to the canonical transcript sequence is represented. Splicing is represented as bold lines and numbers below boxes indicate the exon numbers. Rectangles represent the exons, with 0.25 cm in length being equivalent to 100 nucleotides, and the bold line represents the introns, with 0.5 cm being equivalent to 10 kilobase pairs. Regions of alternatively spliced exons 3 and 36 are lightly shaded. (B) Comparative alignment of nucleotide sequences around exonics splicing sites at exons 3 and 36 of human *CHD7 *with the corresponding exonics regions in Chimpanzee, Horse, Dog, Mouse, short-tailed Opossum, Rat, Boar, Cattle and Chicken. The splicing donor (SD), putative branch point (BP) and splicing acceptor (SA) sites are boxed. The pyrimidine-rich region is shown in bold. Nucleotides differing from the human sequence are underlined.

Re-amplification, cloning and sequencing of the 3.3 kbp DNA band followed by BLAST alignment of its sequence against the human transcripts database revealed that it constituted an alternative transcript of the *CHD7 *gene. Structural analysis of this sequence revealed an alternative exon-intron boundary in this alternative transcript, which was formed by an alternative exonic donor site at exon 3 and an alternative exonic acceptor site at exon 36 (Figure 2a). The sequence of this alternative transcript (CHD7 CRA_e) was deposited at Genbank [Genbank: GU060498]. Sequence analysis of the sequences flanking the alternative splicing sites present in the CHD7 CRA_e transcript revealed the presence of highly conserved sequences associated with splicing (Table 1). In addition, alignment of the sequences flanking the alternative splicing sites in the CHD7 CRA_e alternative transcript with the correspondent exonic regions of the *CHD7 *orthologs found in Genbank revealed a high level of sequence conservation around the putative splicing elements (Figure 2b). In some instances (*Sus scrofa*, *Bos taurus *and *Gallus gallus*), a markedly increased sequence conservation around the alternative splice sites was observed (Figure 2b).

**Table 1:** Analysis of the sequences flanking the alternative splice sites of the CHD7 CRA_e alternative transcript

Alternative transcript	Donor splice site location (nt)^a^	Acceptor splice site location (nt)^a^	Intron size /kb	EXON/intron/EXON (consensus splice sequence)^b^ 5' CAG|guragu...cu/gac/u(y)_10 15_nyag|G) 3'
CHD7 CRA_e	Exon 3 (47)	Exon 36 (112)	81	5' **CAG**|**gu**u**agu**...**c**a**gau**gugcug**uuuuccuauuucag**|A 3'

a) Location of the exonics donor and acceptor splice sites of the CHD7 CRA_e alternative transcript. The numbers in parenthesis represent the exonic splicing sites at exons 3 and 36; b) The consensus sequence associated to splice donor, branch and acceptor sites according to Padgett [30] and Keller and Noon [31]. The vertical lines represent either 5' or 3' splice site and the putative branch point is underlined. The sequence of the investigated splice site in the CHD7 CRA_e transcript is represented below the consensus splice sequence. Nucleotides identity between the consensus and the investigated splice sites are shown in bold r = purine (A or G); y = pyrimidine (C or U)

The *CHD7 *alternative transcript consensus sequence was aligned to the February 2009 version of the human genome sequence assembly provided by the University of California, Santa Cruz (UCSC), using the BLAT search tool to compare the CHD7 CRA_e alternative transcript exon-intron structure with *CHD7 *known mRNA sequences and known spliced ESTs. No human *CHD7 *transcripts or spliced ESTs aligning to the *CHD7 locus *presented an exon-intron structure similar to that displayed by the CHD7 CRA_e alternative transcript (data not shown).

### Analysis of the putative protein generated by the novel CHD7 alternative transcript

**Figure 3:**
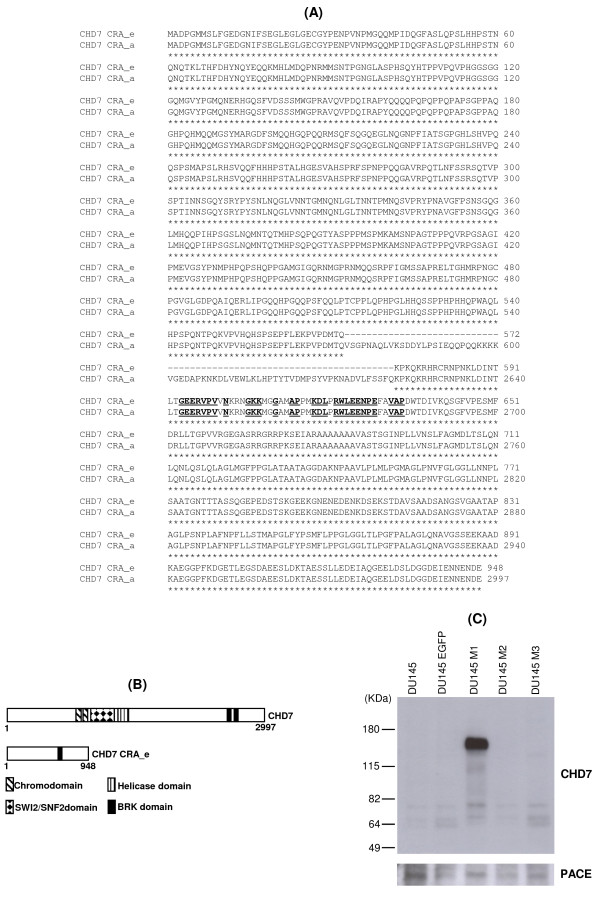
**Deduced protein sequence coded by the CHD7 CRA_e alternative transcript and respective Western blot analysis**. (A) Alignment of the putative amino acids sequence coded by the CHD7 CRA_e alternative transcript with the amino acids sequence of the canonical CHD7 protein (accession number: NP_060250). The amino acids in bold and subtitled are conserved in the BRK domain (smart00592). The amino acids from the canonical CHD7 protein that are missing in the CHD7 CRA_e putative amino acid sequence were omitted for clarity (amino acids 601 to 2580). The numbers on the right refer to the amino acids at the end of each line related to each sequence. (B) Schematic representation of the domain structure of canonical CHD7 protein and CHD7 CRA_e isoform. (C) Western blot analysis of DU145 cells stably expressing CHD7 CRA_e alternative transcript (DU145 M1) using an anti-CHD7 antibody which recognizes the C-terminus end of CHD7. Parental DU145 cells and cells transduced with the empty vector (DU145 EGFP) or stably expressing two additional clones of CHD7 CRA_e alternative transcript, which putatively code for truncated proteins (DU145 M2 and DU145 M3) were used as negative controls. Detection of the PACE protein expression is shown as an internal protein loading control.

The ORFinder tool predicts that the novel CHD7 CRA_e transcript encodes a putative protein of 948 amino acids with a predicted molecular mass of approximately 100 kDa. Alignment of the putative protein encoded by the novel *CHD7 *transcript [GenBank: ACY35999.1] against the canonical CHD7 protein sequence [GenBank: NP_060250] showed that the former lacks most of the central portion and conserves only the N and C-termini of the canonical protein (Figure 3a). Furthermore, a Conserved Domain Database search at NCBI indicated that the putative protein retains only one conserved domain present in the canonical CHD7 protein, namely the BRK domain [GenBank: smart00592] located at its C-terminus (Figure 3b).

To confirm, by Western blot analysis, whether the novel CHD7 CRA_e transcript is in fact translated into a 100 kDa protein, we sub-cloned its coding sequence into the bicistronic lentiviral pLV-EGFP expression vector and overexpressed CHD7 CRA_e isoform in the DU145 prostate carcinoma cell line. This cell line was separately transduced with vector constructions containing the coding sequences of each of all three different clones (CHD7 pLV-M1, CHD7 pLV-M2 and CHD7 pLV-M3) of the novel transcript and, also, with the vector containing only the EGFP coding sequence, as a negative control. Sequencing of these three CHD7 CRA_e cDNA clones (M1, M2 and M3) revealed that M2 and M3 displayed nonsense mutations in their sequences, causing a premature stop-codon and putative truncated proteins at amino acids 718 and 285, respectively. The M1 clone sequencing revealed four missense mutations (M80T, V323A, T408A and K797E). Since the antibody used in this Western blot analysis recognizes the C-terminus end of the CHD7 protein (residues 2,950-2,997 in the canonical protein or residues 901-948 in the novel isoform), the cells transduced with the CHD7 pLV-M2 and CHD7 pLV-M3 expressions constructs were also used as negative controls in addition to the non-transduced DU145 cells.

This Western blot analysis revealed that the novel *CHD7 *transcript is translated into a protein that is specifically recognized by the CHD7 antibody, displaying an apparent molecular mass of 145 kDa (Figure 3c). Immunoprobing of the ubiquitously expressed PACE was employed as the loading control.

### Expression analysis of the CHD7 CRA_e alternative transcript through RT-PCR in normal human tissues

**Figure 4:**
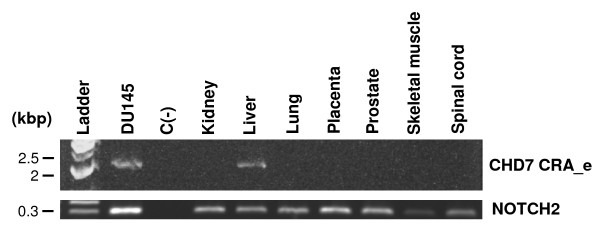
**RT-PCR analysis of the CHD7 CRA_e transcript expression in normal human tissue total RNA samples**. To detect the novel *CHD7 *transcript by RT-PCR we used primers adjacent to the alternative splice site which generate a DNA fragment of approximately 2 kbp. As internal control of the reaction we performed RT-PCR of the same samples using primers specific for the *NOTCH2 *transcript which yield a DNA fragment of approximately 300 bp (lower part of the figure). A negative control without cDNA was run with each reaction.

In order to evaluate whether the novel CHD7 CRA_e transcript is also expressed in normal human tissues besides the DU145 prostate tumoral cell line, we designed an RT-PCR assay to specifically detect its expression, by employing primers which specifically anneal to sequences flanking the alternative splice site of this transcript, generating a DNA fragment of approximately 2 kbp. We used samples of total RNA from normal human tissues (kidney, liver, lung, placenta, prostate, skeletal muscle and spinal cord) from a commercially available total RNA pannel, as described in Methods. DU145 cells total RNA was used as a positive control for the reaction. A negative control, without cDNA, was run with each reaction. In addition to DU145 cells, expression of the novel CHD7 CRA_e transcript was detected only in the liver RNA sample (Figure 4). This result was confirmed using two other primer sets flanking the splice region (data not shown). Analysis of *NOTCH2 *expression was used as an internal control.

## Discussion

Here, we describe a novel alternative transcript of the *CHD7 *gene, which lacks most of its 38 exons, of which 37 are coding. This novel *CHD7 *transcript is likely to be expressed at low abundance and/or in a restricted set of tissues, since no corresponding mRNA or EST has been deposited in public sequence databases to date. Our findings suggest that CHD7 CRA_e is a functionally significant alternative transcript and not an aberrant transcript or splice event associated with a specific cell line or cancer cells. Firstly, we could detect highly conserved sequences associated with splicing events flanking the splice sites of CHD7 CRA_e at exons 3 and 36. Also, a human EST [GenBank: BI039198.1] is available, supporting the fact that the exonic donor site is functional (data not shown). Additionally, splice consensus sequences flanking the splice site of CHD7 CRA_e are highly conserved in various species (Figure 2b). The observed high level of conservation in the sequences of some species proximal to the alternative splice site as well as within the alternatively spliced region related to CHD7 CRA_e splice event indicates that cis-elements may be associated with splicing control in this region. However, considering that the alternative splicing sites are part of the coding region, the conservation observed could also be due to a highly conserved domain in this region. Secondly, detection of the CHD7 CRA_e transcript in normal human liver total RNA, in addition to the DU145 prostate carcinoma cell line, is an indication that this alternative *CHD7 *transcript is also expressed in normal human tissues.

Our molecular analysis predicted that the CHD7 CRA_e alternative transcript encodes a 101 kDa protein. A commercially available antibody raised against the C-terminal end of mouse CHD7 allowed us to detect a distinct protein band with an apparent molecular mass of 145 kDa in the total protein extract of DU145 cells overexpressing the CHD7 CRA_e isoform. Currently, we cannot offer an evidence-based explanation for this discrepancy between the theoretical and the experimentally obtained values for the molecular mass of the CHD7 CRA_e isoform. One possibility is that CHD7 is subject to phosphorylation and/or a myriad of post-translational modifications, similarly to several other helicases and nuclear proteins. If that is the case, CHD7 CRA_e may undergo phosphorylation or other modifications, which could partially explain its different mobility in SDS-PAGE gels. It is interesting to note that the CHD7 CRA_e translated sequence harbors six Serine/Glutamine (SQ) and two Threonine/Glutamine (TQ) motifs, which are known to be putative phosphorylation sites for the DNA damage response kinases, namely: ATM (Ataxia-Telangiectasia Mutated) and ATR (Ataxia-Telangiectasia and Rad3-related) [24]. In fact, proteomic analysis demonstrated that ATM phosphorylates at least one of these motifs [25]. If one can show that these serine/threonine residues are phosphorylated in CHD7 CRA_e, this could partially explain the mobility discrepancy, as well as would implicate CHD7 CRA_e as a putative ATM/ATR substrate, with a possible role in DNA damage response and repair. However, additional possibilities other than post-translational modifications that could account for the CHD7 CRA_e isoform unexpected SDS-PAGE mobility could be a) a particular amino acid composition that could lead to an anomalous migration on SDS-PAGE as it is described for some proteins, for example, the CTCF (CCCTC binding factor) transcription factor [26] and b) an artifact related to the artificial overexpression of CHD7 CRA_e, maybe an aggregate resistant to reduction by 2-mercaptoethanol and/or solubilization by SDS. Additionally, we could not detect endogenous CHD7 CRA_e at the protein level by Western blot, either in total or nuclear-enriched protein extracts obtained from the DU145 cells despite the detection of a protein band of approximately 350 kDa, which most likely corresponds to the full-length CHD7 isoform, and, also, additional protein bands which could represent additional isoforms or degradation products (data not shown). This suggests that the CHD7 CRA_e isoform may be expressed at very low levels at least in DU145 cells.

Analysis of the CHD7 CRA_e predicted protein revealed that only the extreme N-terminal and C-terminal regions of the canonical protein are maintained in this novel isoform (Figure 3b). Thus, CHD7 CRA_e isoform lacks most of the characteristic domains of the CHD7 protein, retaining only one BRK domain in its C-terminal region. The function of the BRK domain is unknown, but it was first described in *Drosophila *CHD7 ortholog KIS protein and is also found in BRM (Brahma) which is another chromatin-remodeling protein [8]. It is hypothesized that the BRK domain may interact with chromatin components, which are unique to higher eukaryotes, since it is not present in yeast chromatin-remodeling factors, such as SWI2/SNF2 and STH1 (SNF Two Homolog 1) [8]. Considering that the chromodomains and ATPase/helicase domains are likely to be critical for the function of the CHD7 protein, the discovery of a CHD7 isoform lacking these domains is quite interesting and intriguing. If this isoform retains the ability to interact with DNA and/or proteins related to chromatin remodeling, it could act as a regulatory protein in this process rather than acting as a helicase, like the full-length protein.

A question that emerges with the description of this novel CHD7 isoform, and probably of other isoforms in the near future, is whether individuals with CHARGE carrying mutations affecting both the full-length and the shorter isoforms differ with respect to clinical features from individuals with mutations affecting only the full-length and not the shorter isoforms. Apparently, pathogenic mutations (stop-codon, frameshift, missense and exon-intron boundary mutations) are scattered throughout the *CHD7 *coding exons in approximately 60% of individuals with CHARGE. Some of the described mutations are on exons 2, 3, 36, 37 and 38 and would also affect the CHD7 CRA_e isoform, but most of the described mutations occur in other exons and would affect only the full-length isoform [1-4]. However, a genotype-phenotype correlation study of *CHD7 *mutation-positive CHARGE individuals revealed no clear correlation between the type of mutation and clinical findings [4]. In fact, different clinical phenotypes were observed either between non-related individuals carrying the same mutation or even between twins, suggesting that additional factors could be involved in CHARGE pathogenesis such as variable chromatin methylation profiles among these individuals caused by differential epigenetic regulation [4].

Finally, the *CHD7 *gene has recently been shown to be involved with the pathogenesis of other diseases, in addition to the CHARGE syndrome. Before the full-length structure of this gene was resolved, it had been described that the protein encoded by KIAA1416, a partial *CHD7 *transcript, is a colon tumor antigen, which is overexpressed in colon cancer cells [27]. Moreover, rearrangements at *CHD7 locus *have recently been associated to small-cell lung cancer [28]. Furthermore, *CHD7 *has been found to be mutated in idiopathic hypogonadotropic hypogonadism and Kallmann syndrome [29]. Altogether, further knowledge on the CHD7 isoforms (including CHD7 CRA_e) and their biological roles should significantly impact our current understanding of several diseases, including developmental disorders and cancer.

## Conclusions

Here we described the exon/intron structure of a novel splicing variant of the human *CHD7 *gene, detected its expression at the mRNA level in at least one human normal tissue (liver) and confirmed that this novel transcript is translated into a protein of apparent molecular mass of 145 kDa. We believe that characterization of the CHD7 CRA_e novel isoform presented here not only sets the basis for more detailed functional studies of this isoform, but, also, contributes to the alternative splicing annotation of the *CHD7 *gene.

## Competing interests

The authors declare that they have no competing interests.

## Authors' contributions

CC designed research, performed the RT-PCR experiment to isolate the *CHD7 *canonical transcript cDNA and revised the manuscript. FST carried out the CHD7 CRA_e alternative transcript sequence and expression analysis (RT-PCR and Western blot) and drafted the manuscript. RGC designed research, isolated and cloned the CHD7 CRA_e alternative transcript cDNA and revised the manuscript. MCS designed research and revised the manuscript. MAAD designed research, carried out CHD7 CRA_e sequence analysis and expression analysis (Western blot) and drafted the manuscript. All authors read and approved the final manuscript.
